# Microbiological Evaluation of Four Different Suture Materials Used for the Surgical Removal of Impacted Lower Third Molars: A Single-Center Prospective Comparative Study

**DOI:** 10.7759/cureus.49370

**Published:** 2023-11-24

**Authors:** Sai Krishna, Rajprakash Bhaskaran, Santhosh P Kumar, Murugesan Krishnan, Saravanan Lakshmanan

**Affiliations:** 1 Oral and Maxillofacial Surgery, Saveetha Dental College and Hospitals, Saveetha Institute of Medical and Technical Sciences, Saveetha University, Chennai, IND

**Keywords:** silk sutures, suture material, impacted lower third molars, postoperative infection, bacterial colonization, surgical extraction, monocryl, vicryl plus, prolene, microbiological evaluation

## Abstract

Introduction

Sutures play a crucial role in the postoperative healing process, as they help approximate wound edges, promote hemostasis, and support tissue healing. The oral cavity harbors a diverse microbial population, and oral surgical procedures can introduce potential pathogens into the surgical site. Understanding the impact of suture material on wound infection rates and the colonization of potentially harmful microorganisms is vital for improving patient outcomes. This study was aimed to evaluate and compare the microbiological properties of prolene, vicryl plus, monocryl, and silk sutures used after the surgical removal of impacted lower third molars.

Materials and methods

A total of 40 patients requiring surgical extraction of impacted lower third molars were assigned to four groups: prolene, vicryl plus, monocryl, and silk sutures. Surgical extraction of impacted tooth was done, and wound was sutured with the abovementioned four different materials in four groups, respectively. After seven days, the sutures were removed and sent to the microbiology lab for colony count assessment. Total microbial colony count, streptococcus count, and lactobacillus count were assessed. Data was analyzed using IBM SPSS Statistics for Windows, Version 23.0 (Released 2015; IBM Corp., Armonk, New York, United States) with p-values less than 0.05 considered as statistically significant. The one-way analysis of variance (ANOVA) and post-hoc Tukey test were done to compare intergroup relations.

Results

The microbiological evaluation of the sutures revealed significant differences in bacterial colonization among the four groups. More bacterial quantities were found in the silk group followed by the monocryl, vicryl plus, and prolene groups in the descending order. Prolene demonstrated the lowest incidence of bacterial growth (p<0.001) compared to vicryl plus, monocryl, and silk sutures. Bacterial colony count was highest in the silk group. The predominant bacterial species found in all groups were *Streptococcus viridans*, *Staphylococcus aureus*, and *Lactobacillus*.

Conclusion

It was found that prolene and vicryl plus sutures exhibited superior microbiological properties compared to monocryl and silk sutures when used for the surgical removal of impacted lower third molars. The lower incidence and less quantity of bacterial colonization on prolene sutures suggest their potential for reducing the risk of postoperative infection; hence, these sutures can be preferred for oral surgical procedures.

## Introduction

Surgical extraction of impacted teeth, particularly lower third molars, is most often performed in oral surgery [1]. The success of such surgeries not only relies on the surgical technique but also depends on the type of suture material used for wound closure [2]. Sutures play a vital role in achieving wound stability, promoting healing, and preventing postoperative complications, including infection [3]. Selection of suture material plays a crucial role as there are various types of sutures available each with distinct characteristics and properties [4]. Vicryl, vicryl plus, monocryl, and silk sutures are frequently utilized in dental surgery, but their comparative microbiological performance has not been extensively studied. Microbial colonization on sutures can contribute to the development of postoperative infections, and bacterial adherence can significantly impact the outcomes and patient's quality of life [5].

In recent years, antibacterial-coated sutures were produced, released onto the market, and utilized which has varied results [6,7]. Triclosan is a halogenated phenol with broad-spectrum activity that finds widespread use in many countries as an additive in disinfectant products, mouthwash, toothpaste, soaps, cloth, deodorant, and shampoo. It works well against a variety of bacterial species and certain types of fungi. It breaks through the cell wall and alters the synthesis of ribonucleic acid (RNA) and other macromolecules in bacteria by targeting a variety of cytoplasmic and membrane receptors which stops the growth of the bacteria and results in cellular death [8]. The effect of triclosan on the rate of inflammation and infection at surgical sites was assessed. Based on the surgical site infection risk factors, a comparison was conducted, and they concluded that using polyglactin 910 sutures can save up to 1.5 million United States dollars (USD) in healthcare resources annually. Because of its antibacterial qualities, triclosan-coated sutures have been demonstrated in recent research conducted in domains other than oral surgery to be a useful tool in lowering the incidence of postoperative infection and bacterial development on the skin [9].

A clinical trial evaluated the antibiotic properties of the combination of iodoform and calendula. On the first and 15th days following the procedure, the suture material was removed, and colony-forming unit (CFU)/ml counts were assessed in case and control groups. Bacterial growth significantly decreased in the case group. It was concluded that the antimicrobial ointment effectively reduces the growth of bacteria on silk sutures which eventually caused the surgical site's irritation to decrease [10]. It has been demonstrated that the antibacterial properties of vicryl suture might negatively impact oral normal flora in addition to being unsuccessful in decreasing the quantity of gram-negative bacteria and its usage is not advised. Extensive advertisement has been done for the vicryl suture materials, and there was limited research regarding the other different types of suture materials [11].

The aim of the study was to evaluate and compare the microbiological performance of prolene, vicryl plus, monocryl, and silk sutures after the surgical extraction of impacted lower third molars. By assessing the extent of bacterial colonization on these suture materials, insights can be gained into their potential to reduce the risk of postoperative infections. The results of this investigation will clarify the possible advantages of using specific suture materials, especially in terms of reducing the risk of postoperative infections, and aid in optimizing patient care in oral surgery. If a type of suture demonstrates superior microbiological performance in terms of reduction in bacterial colonization, it could be recommended in clinical dental practice.

## Materials and methods

Study design and setting

This prospective comparative study was conducted at Saveetha Dental College and Hospitals in Chennai, India, over a duration of four months from May 3, 2023, to October 7, 2023. Ethical committee approval was obtained from the institution (approval number: IHEC/SDC/OMFS-2204/23/154), and informed consent was obtained from all the participants.

Inclusion criteria

A total of 40 participants of age ranging from 20 to 40 years, irrespective of gender requiring surgical extraction of impacted lower right or left third molars with position A class I mesio-angularly impacted teeth, and patients under the American Society of Anesthesiologist's category I were enrolled in the study. 

Exclusion criteria

Patients who are medically compromised like having diabetes and hypertension, pregnant females, anxious patients, patients with a history of allergy to anesthetics, inflammation at the surgical site, and presence of prosthesis in the oral cavity, smokers, and alcoholics were excluded from the study.

Procedure

A total of four groups with 10 samples under each group were allocated using a simple random sampling technique. All the procedures were performed by the same surgeon. Under standard aseptic conditions, the extraoral region was wiped with povidone-iodine, and draping of the patient was done. Inferior alveolar nerve block and lingual and long buccal nerve block were given using local anesthesia (2% lignocaine with adrenaline 1:80,000). The mucoperiosteal flap was raised, buccal bone guttering was done, and the tooth was elevated, luxated, and removed. After achieving hemostasis, suturing was done using prolene, vicryl plus, monocryl, and silk suture materials in the respective groups. Five millimeters of suture was left during cutting. Gauze was placed over the surgical site, and the patient was asked to bite for half an hour and to remove after that period. Aceclofenac and paracetamol combination was prescribed as a pain control medication. The patient was given postoperative instructions, which included not using mouthwash for the first week following surgery, but they were advised to do routine brushing.

**Figure 1:**
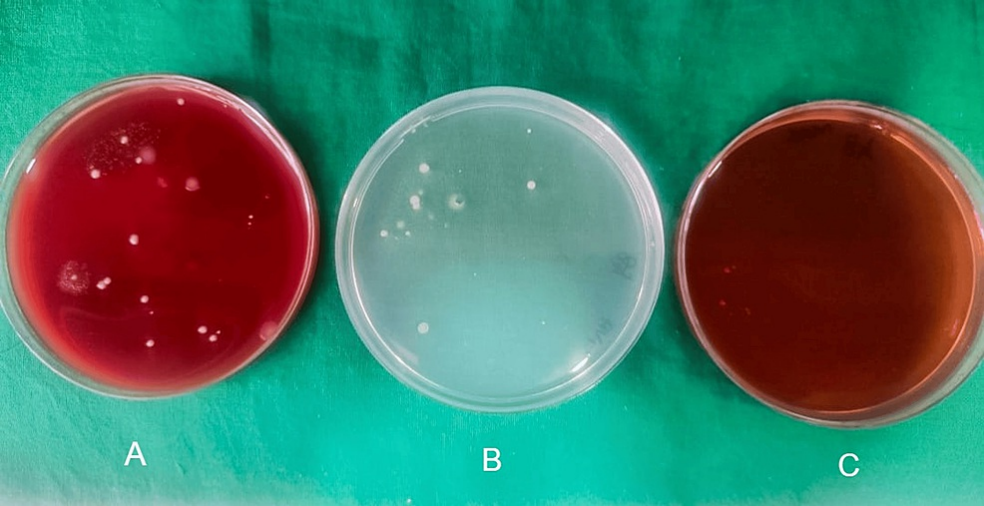
Culture media used for the prolene group A: blood agar culture media; B: MacConkey agar culture media; C: chocolate agar culture media

**Figure 2:**
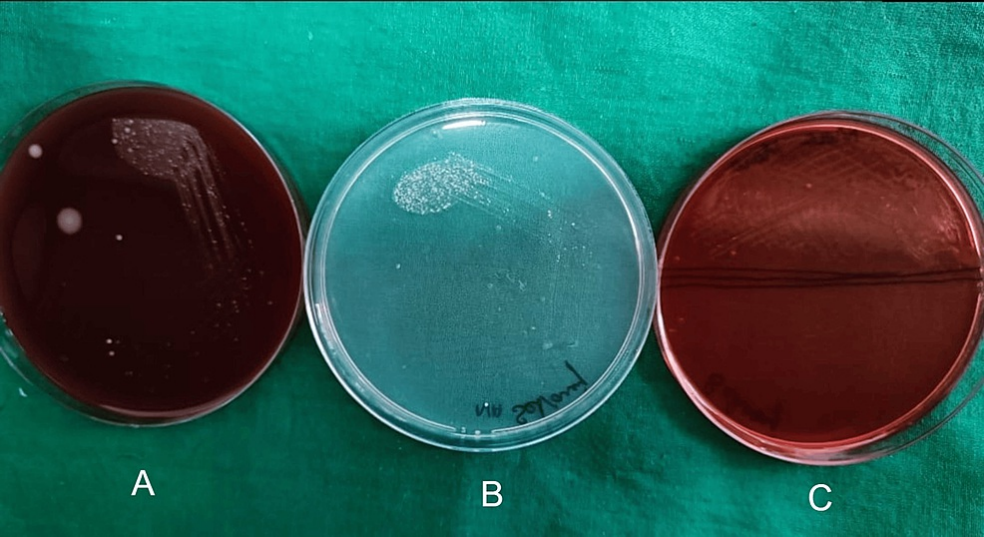
Culture media used for the vicryl plus group A: blood agar culture media; B: MacConkey agar culture media; C: chocolate agar culture media

**Figure 3:**
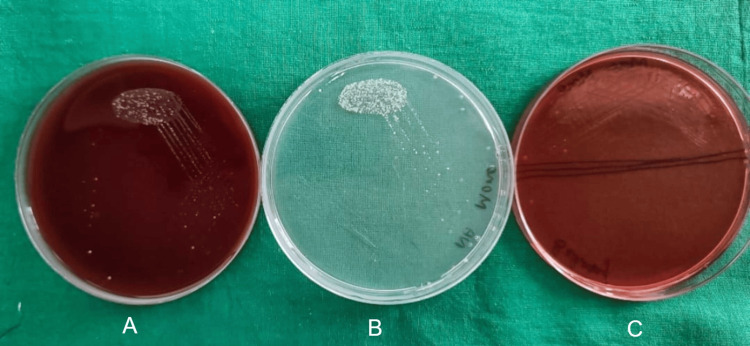
Culture media used for the monocryl group A: blood agar culture media; B: MacConkey agar culture media; C: chocolate agar culture media

**Figure 4:**
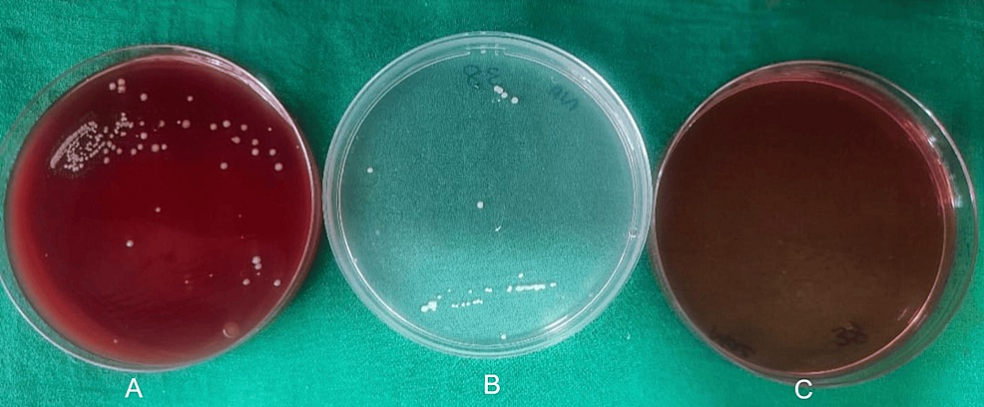
Culture media used for the surgical silk group A: blood agar culture media; B: MacConkey agar culture media; C: chocolate agar culture media

**Figure 5:**
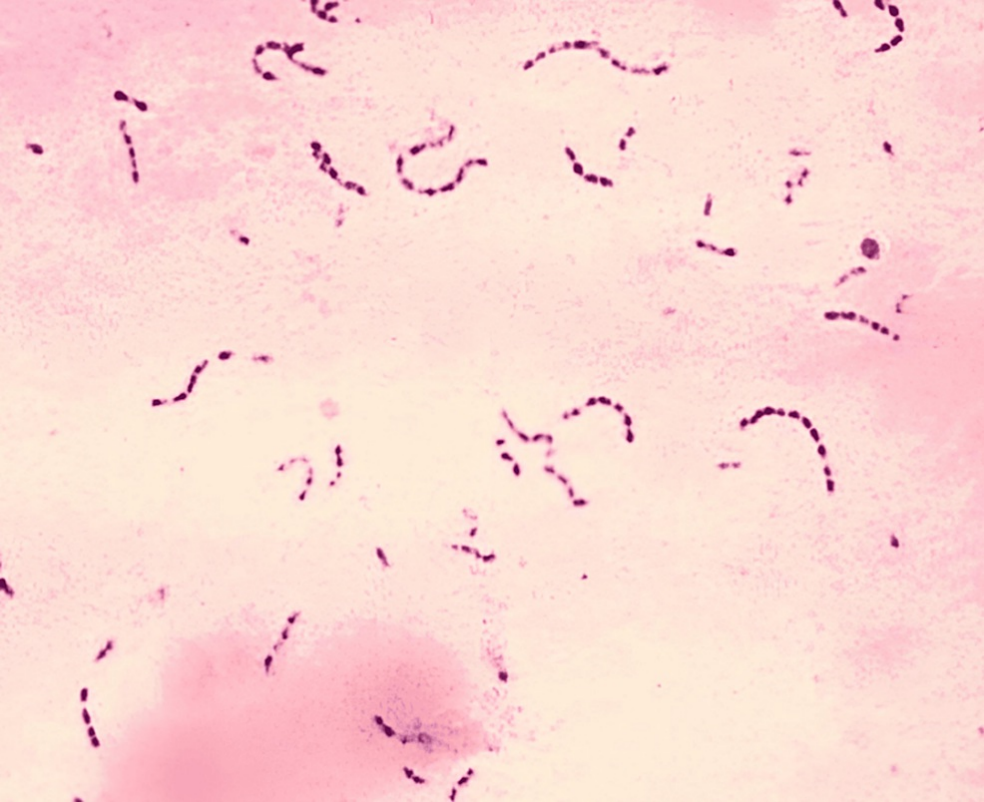
Streptococcus group under optical microscope (100X magnification)

**Figure 6:**
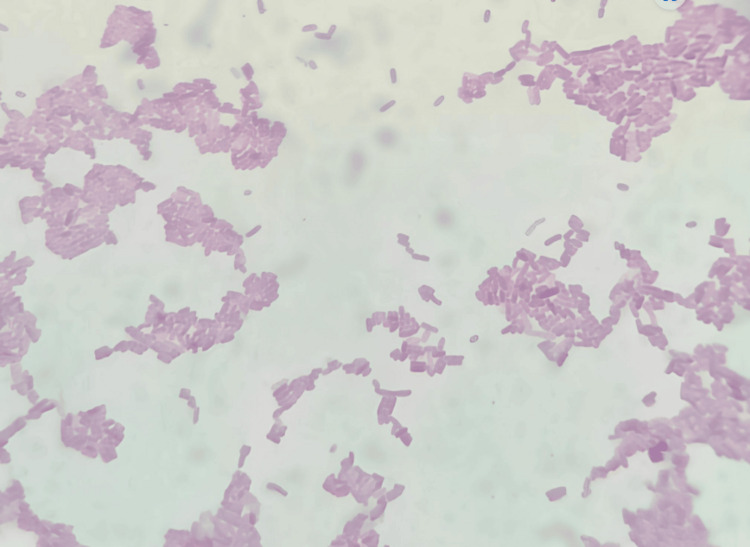
Lactobacillus group under optical microscope (100X magnification)

After seven days, patients were called back and evaluated for surgical site complications such as wound dehiscence and surgical site infection. Irrigation was done and sutures were removed. Sutures were placed in a 5 cc test tube filled with saline. Within one hour of time, the tubes were transported to the microbiology lab. A 10 mm length of the sutures was measured and then transferred to the thioglycolate media and processed. For each sample, one-tenth diluted solution with 0.9% sodium chloride (NaCl) was made. One hundred microliters of it was used on blood agar culture medium, MacConkey agar culture medium, and chocolate agar culture medium, and incubation were done at 37° centigrade for a duration of two days. To obtain the total colony counts, blood agar was used as culture media, chocolate agar was used to culture *Streptococcus* species, and MacConkey agar was used to culture *Lactobacillus* species. Bacterial colonies' growth was observed in the aforementioned three culture media for the prolene suture group (Figure 1), vicryl plus suture group (Figure 2), monocryl suture group (Figure 3), and silk suture group (Figure 4). Gram staining was done, and total colony counts, streptococcus counts (Figure 5), and lactobacillus counts (Figure 6) were assessed under optical microscope. To standardize the mean among all the groups, the colonies were divided into the length of suture material (CFU-length ratio).

Statistical analysis

The normality of the data was assessed by the Shapiro-Wilk test which revealed normal distribution of the data, and thus, parametric tests were done. Data was analyzed using IBM SPSS Statistics for Windows, Version 23.0 (Released 2015; IBM Corp., Armonk, New York, United States) with p-values less than 0.05 considered as statistically significant. The one-way analysis of variance (ANOVA) and post-hoc Tukey test were done to compare intergroup relations.

## Results

**Figure 7:**
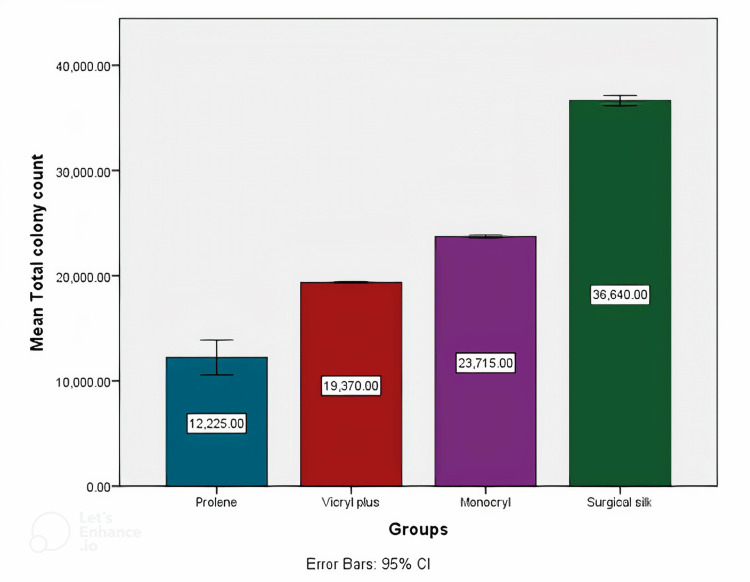
Mean total microbial colony counts among the four different suture material groups

**Figure 8:**
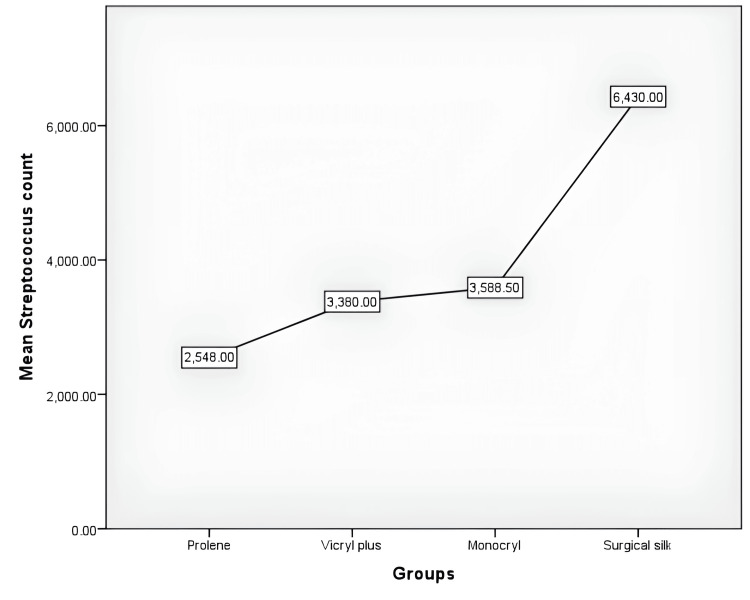
Mean streptococcus counts among the four different suture material groups

**Figure 9:**
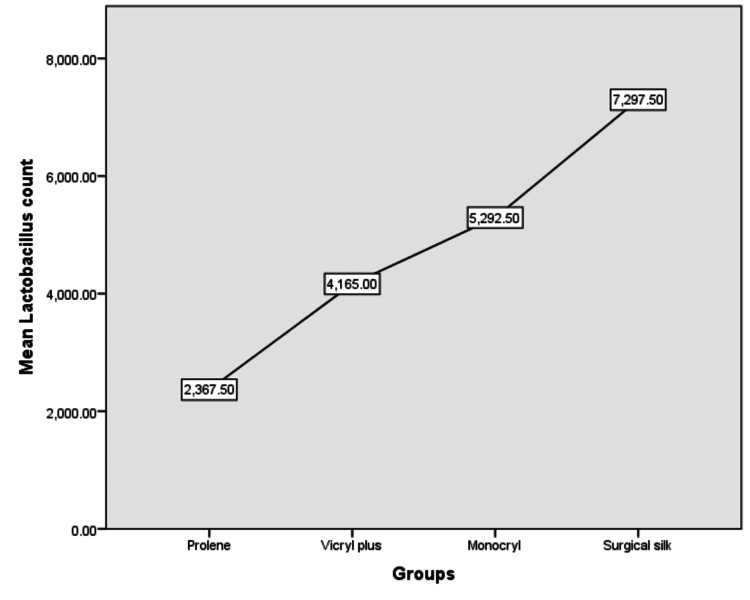
Mean lactobacillus counts among the four different suture material groups

There were 40 patients participating in the study who underwent mandibular third molar extractions. With an equal allocation ratio, the participants were divided into four groups, namely, surgical silk, vicryl plus, monocryl, and prolene. Descriptive statistics revealed that surgical silk had the highest mean total CFU followed by monocryl, vicryl plus, and prolene (Figure 7). Mean streptococcus counts were highest in the silk group followed by the monocryl, vicryl plus, and prolene groups (Figure 8). Mean lactobacillus counts were also highest in the silk group followed by the monocryl, vicryl plus, and prolene groups (Figure 9). The prolene group had the least CFU in total colony count, streptococcus count, and lactobacillus count, while the silk group had the highest CFU in total colony count, streptococcus count, and lactobacillus count.

The one-way ANOVA and post-hoc Tukey test were performed to analyze the differences in the CFU among the groups. The one-way ANOVA (Table 1) reveals that there is a statistically significant difference between the groups in total colony count, and the post-hoc Tukey test (Table 2) reveals a statistically significant difference among the groups with prolene demonstrating the lowest count and surgical silk depicting the highest count.

**Table 1:** One-way ANOVA depicting the total colony count between the groups ANOVA: analysis of variance; **: statistically significant

ANOVA				
	Sum of squares	Mean square	F	Sig.
Between groups	3158377250.000	1052792416.667	711.994	.001**
Within groups	53231500.000	1478652.778		
Total	3211608750.000			

**Table 2:** Post-hoc Tukey test depicting the difference in the distribution of total colony count between the groups p=0.001*: the mean difference between all the groups was statistically significant

Multiple comparisons			
(I) Groups	(J) Groups	Mean difference (I-J)	Sig.
				Prolene	Vicryl plus	-7145.00000	.001*
Monocryl	-11490.00000	.001*		
Surgical silk	-24415.00000	.001*		
Vicryl plus	Prolene	7145.00000	.001*
	Monocryl	-4345.00000	.001*
	Surgical silk	-17270.00000	.001*
Monocryl	Prolene	11490.00000	.001*
	Vicryl plus	4345.00000	.001*
	Surgical silk	-12925.00000	.001*
Surgical silk	Prolene	24415.00000	.001*
	Vicryl plus	17270.00000	.001*
	Monocryl	12925.00000	.001*

The one-way ANOVA (Table 3) reveals that there is a statistically significant difference between the groups in streptococcus count, and the post-hoc Tukey test (Table 4) reveals a statistically significant difference among the groups with prolene demonstrating the lowest count and surgical silk depicting the highest count.

**Table 3:** One-way ANOVA depicting streptococcus count between the groups ANOVA: analysis of variance; ***: statistically significant

ANOVA				
	Sum of squares	Mean square	F	Sig.
Between groups	85662206.875	28554068.958	4658.456	.001***
Within groups	220662.500	6129.514		
Total	85882869.375			

**Table 4:** Post-hoc Tukey test depicting the difference in the distribution of streptococcus count between the groups p=0.001*: the mean difference between all the groups was statistically significant

Multiple comparisons				
(I) Groups	(J) Groups	Mean difference (I-J)	Std. error	Sig.
					Prolene	Vicryl plus	-832.00000	35.01289	.001*
Monocryl	-1040.50000	35.01289	.001*		
Surgical silk	-3882.00000	35.01289	.001*		
Vicryl plus	Prolene	832.00000	35.01289	.001*
	Monocryl	-208.50000	35.01289	.001*
	Surgical silk	-3050.00000	35.01289	.001*
Monocryl	Prolene	1040.50000	35.01289	.001*
	Vicryl plus	208.50000	35.01289	.001*
	Surgical silk	-2841.50000	35.01289	.001*
Surgical silk	Prolene	3882.00000	35.01289	.001*
	Vicryl plus	3050.00000	35.01289	.001*
	Monocryl	2841.50000	35.01289	.001*

The one-way ANOVA (Table 5) reveals that there is a statistically significant difference between the groups in lactobacillus count, and the post-hoc Tukey test (Table 6) reveals a statistically significant difference among the groups with prolene demonstrating the lowest count and surgical silk depicting the highest count. The results show that prolene has the most antibacterial action followed by vicryl plus and monocryl with surgical silk depicting the least antibacterial action.

**Table 5:** One-way ANOVA depicting lactobacillus count between the groups ANOVA: analysis of variance; ***: statistically significant

ANOVA				
	Sum of squares	Mean square	F	Sig.
Between groups	127988421.875	42662807.292	11509.966	.001***
Within groups	133437.500	3706.597		
Total	128121859.375			

**Table 6:** Post-hoc Tukey test depicting the difference in the distribution of lactobacillus count between the groups p=0.001*: the mean difference between all the groups was statistically significant

Multiple comparisons				
(I) Groups	(J) Groups	Mean difference (I-J)	Std. error	Sig.
					Prolene	Vicryl plus	-1797.50000	27.22718	.001*
Monocryl	-2925.00000	27.22718	.001*		
Surgical silk	-4930.00000	27.22718	.001*		
Vicryl plus	Prolene	1797.50000	27.22718	.001*
	Monocryl	-1127.50000	27.22718	.001*
	Surgical silk	-3132.50000	27.22718	.001*
Monocryl	Prolene	2925.00000	27.22718	.001*
	Vicryl plus	1127.50000	27.22718	.001*
	Surgical silk	-2005.00000	27.22718	.001*
Surgical silk	Prolene	4930.00000	27.22718	.001*
	Vicryl plus	3132.50000	27.22718	.001*
	Monocryl	2005.00000	27.22718	.001*

## Discussion

In this study, it was found that there was a significant reduction in the total colony count, streptococcus count, and lactobacillus count with the prolene group, followed by the vicryl plus and monocryl groups. There are four main stages of wound healing which initially starts with inflammation followed by cell proliferation, matrix deposition, and finally tissue remodeling [12]. Tissue approximation is preserved by sutures until the incision reaches a tensional strength that stops it from dehiscing. Tight closure, securing, and stabilizing of the wound margins will aid in the success of surgical treatment [13]. In oral surgery, the behavior of sutures varies depending on the microbiota, salivary flow, and tissue quality. They serve as a conduit for information between the tissues' interior and external areas, which affects wound healing [14].

Some surgeons prefer using silk, a natural, braided material that is non-absorbable. It provides good tension and stability during the suture process [15]. Conversely, the silk suture's braided structure facilitates the collection of debris and bacterial accumulation which leads to inflammation at the surgical site. Prolene, often known as polypropylene suture, is a synthetic monofilament, non-absorbable suture with a high tensile strength. Prolene sutures are made of an isotactic crystalline stereoisomer of polypropylene and are meant to endure for a long time [16]. Polyglactin 910 commercially known as vicryl is a synthetic suture material which is absorbable and is often braided. In tissue, the suture maintains its tensile strength for around two to three weeks before being totally dissolved by acid hydrolysis in eight to 10 weeks. Vicryl and other polyglycolic acid sutures can also be impregnated with triclosan (vicryl plus) to offer antibacterial protection of the suture line, or they can be treated for faster breakdown (vicryl rapid) in tissues that heal quickly, such as mucous membranes [17]. Monocryl is an absorbable, synthetic suture material usually composed of poliglecaprone which is a copolymer of epsilon-caprolactone and glycolide and is available as dyed (violet) and undyed (clear) forms [18]. It has a low tissue reactivity, a half-life of seven to 14 days, and a high tensile strength.

It is commonly known that the risk of infection is increased when the suture material is present inside the tissue. This effect is particularly noticeable when using multifilament materials. Several investigations have shown that when compared to multifilament sutures, monofilament sutures have lesser inflammation at the surgical site. Through capillary action, multifilament sutures encourage bacterial adhesion to other sterile sites, intensifying the infection process [19]. However, because monofilaments are harder to work with and possess sharp ends that irritate the oral mucosa, many physicians prefer multifilament. Additionally, it has been shown that the structure and type of suture used determine wound infection [20].

In this study, it was found that the colony counts of the prolene were less compared to that of the other groups. Although prolene is not having much of antibiotic properties, it was found that the monofilament character of this material led to low colony counts around the suture material. In a study conducted by Otten et al. [21] on the bacterial colonization of non-absorbable monofilament and absorbable multifilament sutures in 11 patients, it was found that the microbial colony counts were higher with the multifilament group than the monofilament group. According to Banche et al., absorbable monocryl had a substantially lower microbial load than non-absorbable multifilament sutures, which were referred to as Supramid (B. Braun, Melsungen, Germany). Polyglactin 910 and silk (multifilament sutures) have shown severe foreign body reaction in comparison to that of polypropylene and poliglecaprone 25 monofilament sutures [22].

Apart from the type of filament, the absorbable property of the suture material has a role to play in the growth of bacterial colonies [23,24]. Compared to non-absorbable materials, which only result in a blind inflammatory response, absorbable sutures may generate a higher degree of inflammation due to their metabolism [25]. According to Kim et al., since the oral cavity is wet and susceptible to infection from saliva, food particles, and bacteria, a suture placed there is affected differently than a suture material placed outside of the mouth [19]. Compared to that of the monocryl group, vicryl plus had shown a smaller number of colony counts that was mainly attributed with the effect of triclosan which was the key ingredient in vicryl plus. The results of the meta-analysis conducted by Guo et al. showed that the triclosan-coated group (vicryl plus) had lesser chances of surgical site infection compared to non-coated sutures which were statistically significant [26].

The effectiveness of vicryl plus in reducing postoperative infections was found in the meta-analysis of Edmiston et al., which included 3568 patients and 13 randomized controlled trials (RCTs) [6]. A similar type of results was reported by Guo and associates which included 17 meta-analyses on 5268 patients [26]. Storch et al. have shown that there was marked reduction in colony counts of *S. aureus* species by 96.7% after a duration of 48 hours at the surgical site [27], and a trial by Ming et al. reported a decrease in *Escherichia coli *and *S. aureus* species [28]. According to a study, it was found that the infection rate was reduced by 87% in patients when vicryl plus was used as the suture material [29]. Marco et al. in their studies found that there was 66% reduction of *Staphylococcus epidermidis* [30]. All these studies strongly correlate to the results that were found in the current study.

From our study results, it was evident that the microbial colony counts, i.e., total colony counts, streptococcus counts, and lactobacillus counts, around the prolene suture were less compared to that of the vicryl plus, monocryl, and surgical silk and the difference between the groups was statistically significant (p=0.001). It can be attributed to the properties of the material as prolene is unbraided, thereby leading to less plaque formation around the suture material. Prolene is most often used as an extraoral suture material, and there are limited studies of prolene as an intraoral suture material. Therefore, the usage of prolene as an intraoral suture material has to be tried on large samples, because intraorally the presence of saliva plays a huge role in microbial adhesion to the suture materials.

Limitations of the study

Properties of suture materials might vary at different anatomical sites in the oral cavity which were not evaluated in this study. The study was conducted at the single center, and the microbial flora in the different populations might vary. Hence, multi-centric study on a larger sample size with other microbial colony counts must be evaluated.

## Conclusions

It was found that prolene and vicryl plus sutures exhibited superior microbiological properties compared to monocryl and silk sutures when used for the surgical removal of impacted lower third molars. The lower incidence and less quantity of bacterial colonization on prolene sutures suggest their potential for reducing the risk of postoperative infection; hence, these sutures can be preferred for oral surgical procedures.
